# Environmental Adaptation of Genetically Uniform Organisms with the Help of Epigenetic Mechanisms—An Insightful Perspective on Ecoepigenetics

**DOI:** 10.3390/epigenomes7010001

**Published:** 2022-12-26

**Authors:** Günter Vogt

**Affiliations:** Faculty of Biosciences, University of Heidelberg, Im Neuenheimer Feld 234, 69120 Heidelberg, Germany; gunter.vogt@web.de

**Keywords:** asexual populations, epigenetic ecotypes, ecoepigenetics, DNA methylation, environmental adaptation, general-purpose genotype, invasion paradox, phenotypic plasticity

## Abstract

Organisms adapt to different environments by selection of the most suitable phenotypes from the standing genetic variation or by phenotypic plasticity, the ability of single genotypes to produce different phenotypes in different environments. Because of near genetic identity, asexually reproducing populations are particularly suitable for the investigation of the potential and molecular underpinning of the latter alternative in depth. Recent analyses on the whole-genome scale of differently adapted clonal animals and plants demonstrated that epigenetic mechanisms such as DNA methylation, histone modifications and non-coding RNAs are among the molecular pathways supporting phenotypic plasticity and that epigenetic variation is used to stably adapt to different environments. Case studies revealed habitat-specific epigenetic fingerprints that were maintained over subsequent years pointing at the existence of epigenetic ecotypes. Environmentally induced epimutations and corresponding gene expression changes provide an ideal means for fast and directional adaptation to changing or new conditions, because they can synchronously alter phenotypes in many population members. Because microorganisms inclusive of human pathogens also exploit epigenetically mediated phenotypic variation for environmental adaptation, this phenomenon is considered a universal biological principle. The production of different phenotypes from the same DNA sequence in response to environmental cues by epigenetic mechanisms also provides a mechanistic explanation for the “general-purpose genotype hypothesis” and the “genetic paradox of invasions”.

## 1. Introduction

The adaptation of organisms to different environments requires phenotypic diversity, which can be generated by genetic mechanisms such as mutation and recombination or by different expression of the same genome in response to different environmental cues. The latter possibility is called phenotypic plasticity [1,2,3,4]. Phenotypic plasticity occurs in all organisms, but seems to be particularly important for asexually reproducing animal and plant populations and species with longer generation times, in which genetic recombination is absent and the mutation frequency per time unit relatively low, respectively. There is increasing evidence that epigenetic mechanisms such as DNA methylation, histone modifications and non-coding RNAs are among the molecular pathways underpinning phenotypic plasticity [5,6,7,8,9].

Many authors have already written on the potential importance of epigenetic mechanisms for environmental adaptation, e.g., [10,11,12]. However, previous research in this field was conducted with relatively small fragments of the genome, and, therefore, the claimed role of epigenetic mechanisms in the production of phenotypic variation was often doubted [13]. More meaningful analysis on the whole-genome scale in statistically relevant numbers of individuals was hampered by the limited sensitivity of the available methods, the need of large amounts of tissue per measurement often requiring pooling of different tissues and specimens, and very high costs. The development of affordable, highly sensitive and fast genetic and epigenetic approaches, such as third generation sequencing [14] and whole genome bisulfite sequencing [15] that require only small tissue samples, made it possible to investigate the epigenetics–environmental adaptation relationship in more detail. Examples from different kingdoms of life are discussed below, focussing on genetically uniform populations.

Asexually reproducing organisms can inhabit very broad ranges of habitats and geographical regions and stably adapt to them despite the scarcity or virtual absence of genetic variation [16,17,18], which was often explained by the existence of so-called “general-purpose genotypes” [19,20,21]. Likewise, small invasive groups can be very successful in new environments despite paucity of genetic variation, which is known as the “genetic paradox of invasions” [22,23]. The molecular mechanisms underlying these phenomena are largely unknown but epigenetic mechanisms are prime candidates.

The adjustment of organisms to their environment is usually described by one of the two terms “acclimation” and “adaptation” [24]. Acclimation is the short and medium- term adjustment that is based on phenotypic plasticity, the ability of a genotype to produce different phenotypes in response to different environmental cues [25]. The induced changes can be biochemical, physiological, behavioural and morphological and are principally reversible. In contrast, evolutionary adaptation, mostly abbreviated as adaptation, is an irreversible long-term process spanning over many generations that requires genetic variation, fitness enhancement and selection. Most populations discussed in this paper are genetically uniform and live in their habitats for several decades, fitting better to acclimation than adaptation. Because it cannot be excluded that some of them are already on the way to evolutionary adaptation, I will here use the more neutral term “environmental adaptation” to avoid confusion.

This review paper examines the role of epigenetic mechanisms in environmental adaptation of genetically uniform populations. It starts with a comparison of the production of phenotypic diversity by genetic and epigenetic mechanisms. Thereafter, several case studies are presented that show close associations between epigenetic signatures and environmental adaptation in asexually reproducing populations and genetically depauperate invasive groups. Finally, the relevance of epigenetically mediated environmental adaptation is examined for genetically uniform animals, plants and microorganisms, with a brief digression into sexually reproducing species. Also discussed is the suitability of epigenetic adaptation to explain the “general-purpose genotype hypothesis” and the “genetic paradox of invasions”.

## 2. Generation of Phenotypic Diversity in Populations

The phenotypic diversity required for environmental adaptation can be produced by genetic and epigenetic mechanisms (Table 1). The genetic mechanisms include mutation, recombination, drift and gene flow and generate phenotypic diversity by changing the DNA sequence or their relative distribution in populations. Epigenetic mechanisms produce phenotypic diversity by differential expression of the same DNA and do not change the DNA sequence. They include DNA methylation, histone modifications, non-coding RNAs, Polycomb/Trithorax group proteins, chemical mRNA modifications and mRNA editing. Alternative splicing is a special case that produces different mRNAs from the same DNA sequence. It includes genetic and epigenetic components.

### 2.1. Generation of Phenotypic Variation by Genetic Mechanisms

Genetic mutations such as single nucleotide substitutions, duplications, deletions and transversions are fundamental processes for the generation of phenotypic diversity. Random single nucleotide substitutions have a frequency of 10^−8^–10^−9^ per locus and generation for most organisms [26]. The genetic diversity that can be generated by this mechanism is very much dependent on population size and generation time. Populations with low individual numbers and long generation times can produce much less genetic variation than populations with high individual numbers and short generation times. Since most mutations are deleterious or neutral only a smaller proportion contributes to phenotypic variation in a population. Previously, genetic mutations were thought to occur randomly throughout the genome, but this has been challenged in recent years. For example, in the model plant *Arabidopsis thaliana*, mutations were less frequently found in functionally constrained regions of the genome [27]. In gene bodies the mutation rate was reduced by half and in essential genes by two-thirds.

Recombination, the exchange of DNA between maternal and paternal chromosomes during meiosis that produces new allele combinations and phenotypic variants, is typical of sexually reproducing animals and plants. It is variable between higher taxa and species, variable across the genome, and highest under conditions of random mating [28]. The effects of recombination can be positive, facilitating environmental adaptation, or negative, breaking apart beneficial allele combinations. Only sexually reproducing organisms can use recombination to produce phenotypic variation in populations. There are also possibilities of horizontal gene transfer in bacteria, which are summarized under the term recombination [29].

Genetic drift, the random change in the frequency of an allele from generation to generation, may influence the spectrum of phenotypic diversity in a population by either causing gene variants to disappear or causing initially rare alleles to become frequent. The role of drift is expected to be particularly important in small and isolated populations [30]. Gene flow, the transfer of genetic material from one population to another, can also considerably influence phenotypic diversity in a population [31].

### 2.2. Generation of Phenotypic Variation by Epigenetic Mechanisms

The generation of phenotypic diversity from the same genome by epigenetic mechanisms is most easily investigated by performing experiments with genetically identical clone members [32,33]. Suitable animal models are monozygotic twins and polyembryonic multiples of mammals, clone-mates of apomictic parthenogenetic vertebrates and invertebrates, and genets of colonial corals. Good plant models are individuals of clonal lineages and cuttings of the same individual, and good microbial models are yeast and bacterial colonies originating from a single founder cell by binary fission.

The best investigated epigenetic mechanisms that are known to be involved in the generation of phenotypic diversity from the same genome are DNA methylation, histone modifications and non-coding RNAs [34,35,36]. Recent reviews of epigenetic mechanisms in animals, plants, fungi and bacteria are provided by Vogt [37], Maeji and Nishimura [38], Madhani [39] and Sánchez-Romero and Casadesús [40], respectively. The full range of epigenetic chromatin modifications in an organism that can change gene expression and contribute to phenotypic variation is currently unknown. However, the diploid human epigenome contains >10^7^ CpG dinucleotides and >10^8^ histone tails, providing an enormous number of potentially modifiable sites [41]. The number of human microRNA genes, each of which can produce numerous transcripts involved in the generation of phenotypic variation is approximately 2600 [42].

DNA methylation is the best investigated epigenetic mechanism. It is widespread in prokaryotes and eukaryotes but has been lost in some species and groups [37,43]. In animals, the methylation marks are mostly on the cytosines of CpG dinucleotides and occur in genic and intergenic regions including promoters, gene bodies and repeats [34,44,45]. Methylation of promoters and transposons usually results in transcriptional repression, whereas gene body methylation modifies the accessibility of genes in the chromatin, modulates gene expression, and prevents spurious transcription initiation [45,46,47]. The DNA methylation marks in animals are established by DNA methyltransferases (DNMTs) and erased by ten-eleven-translocation enzymes (TETs) [48,49].

In plants, DNA methylation is found in CG, CHG and CHH sequence contexts, where H is A, C or T [50]. Methylation is highly enriched in repressed transposable elements and repeats. Plants have different methylation and demethylation enzymes when compared to animals [50]. In fungi, DNA methylation is mainly found in transposable elements, promoter regions and repetitive DNA sequences [51,52]. It is associated with silencing of gene expression and transposons and is involved in a wide range of biological phenomena including phenotypic switching. Bacterial genomes mainly display adenine methylation, which helps regulating numerous cellular processes such as chromosome replication, correction of DNA mismatches and transcription [53]. It is further involved in bacterial defence and virulence and fosters formation of phenotypically different epigenetic lineages.

Post-translational histone modifications are typical of all eukaryotes [35,54]. The histones in the nucleosomes greatly influence DNA transcription by either shielding the DNA or allowing binding of transcription factors to the DNA. In animals, the N-terminal tails of the histones carry modifications such as methylation, acetylation, phosphorylation and ubiquitination, which affect the chromatin structure. Histone acetylation often stimulates gene expression, whereas histone methylation often represses gene expression, depending on the amino acid residue being modified. The histone modifications are produced by a broad array of enzymes and read by various proteins [55,56]. For example, histone acetylation marks in animals are written by histone acetyltransferases (HATs) and read by bromodomain-containing proteins (BrDs). Information on histone modifications and their regulation in plants and fungi is found in Zhao et al. [57] and Brosch et al. [58], respectively.

Non-coding RNAs are further crucial regulators of gene expression and contributors to phenotypic variation that occur in eukaryotes and prokaryotes [36,59]. They include several classes that differ in sequence length and molecular configuration. In animals, microRNAs inhibit translation or cause mRNA degradation [60,61]. Small interfering RNAs regulate gene transcription through transposable element silencing and the interaction with DNA methylation and histone modifications [62]. Piwi-interacting RNAs mainly silence transposable elements in the germ line at the transcriptional and post-transcriptional levels [63]. Long ncRNAs are involved in transcriptional regulation, dosage compensation and genomic imprinting in mammals and development, insecticide resistance and anti-viral defence in insects [64,65]. Information on the role of non-coding RNAs in plants, fungi and bacteria is found in Waititu et al. [66], Dhingra [67] and Stav et al. [68], respectively.

Polycomb group (PcG) and Trithorax group (TrxG) proteins contribute significantly to the mitotic and meiotic inheritance of epigenetically mediated phenotypic variability in eukaryotes by sustaining silent and active gene expression states through cell generations and the germ line [69]. Ciabrelli et al. [70] demonstrated for isogenic lines of the fruit fly *Drosophila melanogaster* how chromatin organization and PcG proteins support epigenetically heritable phenotypic plasticity.

Phenotypic variation unrelated to genetic variation can additionally be produced by alternative splicing, RNA editing and chemical modifications of the mRNA. Alternative splicing generates multiple transcripts from a single gene. It is basically a genetic mechanism [71], but epigenetic mechanisms can be involved as well. For example, methylation of CpGs and histone modifications can mark an alternative exon, and these marks are then recognized by an adaptor protein that recruits the splicing factors [72]. An example of mRNA editing is the deamination of adenosine to inosine by the ADAR (adenosine deaminase acting on RNA) enzyme family, which can lead to codon change [73]. Chemical modifications of the mRNA can result in codon change and diversification of the proteome and phenome as well [74].

### 2.3. Stochastic and Environmentally-Induced Epimutations and Related Phenotypic Change

Changes of the epigenetic marks on the DNA and histones that can trigger phenotypic variation occur spontaneously or by environmental induction [32,33,75,76,77]. These changes are called epimutations [78]. Epimutations do not alter the DNA sequence and are principally reversible. Like genetic mutations, stochastic epimutations affect first only single individuals of a population. In genetically identical populations, all stochastic epimutations together can generate a phenotypic spectrum around the target or mean phenotype [79,80]. When the environment changes, one of these alternative phenotypes may become the optimal one. Thus, the a-priori production of a spectrum of phenotypic variants from the same genotype by stochastic epimutations without knowing the future conditions can be regarded as a bet-hedging strategy that secures populations against unforeseen changes of the environment. In contrast, environmentally induced epimutations can affect many population members simultaneously, strengthening adaptation to the prevailing conditions and creating phenotypic stability. In wild populations, both sources of epigenetic variation occur together [80] as will be discussed in more detail below.

Stochastic epimutations can be neutral, detrimental or beneficial such as genetic mutations. However, they are several orders of magnitude more frequent than genetic mutations. For example, in the model plant *Arabidopsis thaliana*, stochastic epimutations occur at a rate of 10^−4^ per base pair and generation, whilst genetic mutations occur only at a rate of 10^−9^ [81].

**Figure 1:**
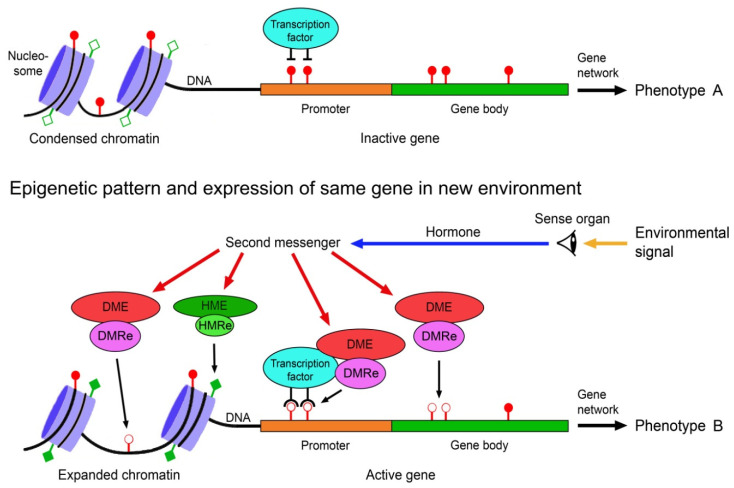
Scheme of environmentally induced change of gene and phenotype expression by epigenetic mechanisms. Environmental signals trigger gene expression change via hormones, second messengers, and environment-sensitive DNA methylation modifying enzymes (DME) and histone modifying enzymes (HME). DNA methylation readers (DMRe), histone modification readers (HMRe) and transcription factors recruit the DMEs and HMEs to specific sites in the chromatin and DNA. Histone modifications such as acetylation (filled squares) and deacetylation (open squares) help to shape chromatin structure and access to the DNA, and methylation (filled circles) and demethylation (open circles) of CpG dinucleotides in the DNA modify gene expression, resulting in different variants of a phenotypic trait. Adapted from Vogt [9].

The environmental induction of phenotypic change via epigenetically-mediated differential gene expression is triggered by stronger and longer lasting environmental cues such as temperature, drought, light, salinity, food, predator odours, injury, toxicants or disease agents [82,83,84]. It requires signal transmission from the external world to the nucleus of the target cells, environment-sensitive molecules involved in gene regulation that can perceive and interpret these signals, readers and editors of epigenetic marks, and molecules that recruit the epigenetic modifiers to specific regions of the DNA and chromatin. These various components must crosstalk to specifically change expression of a gene (Figure 1).

The transmission of environmental signals to the target cells mostly occurs via signal perceiving sense organs and signal transmitting neurohormones and second messengers, which eventually regulate the molecules involved in chromatin remodelling, gene expression and processing of the transcripts. Serotonin is a good example of an environmental signal transmitting hormone. In locust polyphenism, the density-dependent change of morphologically and behaviourally different stationary and migratory phases, it regulates the alternative expression of density-responsive genes with the help of epigenetic mechanisms [85].

A considerable number of proteins participating in the regulation of chromatin architecture and gene expression are responsive to environmental cues. Examples are the DNA demethylating TET, which is up- or downregulated by several environmental factors including food ingredients, ethanol, air pollution and radiation [86], proteins of the Polycomb group that are sensitive to the environmental temperature [87], and transcription factors of the TCP family in vascular plants that mediate environmental signals into growth responses [88].

The writers and erasers of the DNA methylation marks include the methylating DNMTs and the demethylating TETs [48,49]. These enzymes form complexes with readers of the DNA methylation marks such as proteins of the methyl-CpG-binding domain family (MBDs) and transcription factors to exert their functions [89,90,91]. The MBDs bind methylated CpG dinucleotides and act as translators between DNA methylation and histone modifications [91]. Transcription factors with different sequence specificity can guide the methylation modifying molecular complex to specific sites of the DNA [92]. Each animal and plant possess hundreds of such transcription factors.

## 3. Environmental Adaptation of Clonal Organisms with the Help of Epigenetic Mechanisms

Contrary to expectation, many asexually reproducing species and otherwise clonal lineages can adapt to a wide range of geographical latitudes, altitudes and habitats. This phenomenon is often explained by the existence of so-called “general-purpose genotypes”. This section examines if epigenetic mechanisms are involved in the environmental adaptation of clonal populations and if they can mechanistically explain the general-purpose genotype.

### 3.1. Case Studies with Animals

Liew et al. investigated the association of DNA methylation and phenotypic trait variation in differently held laboratory populations of the colonial coral *Stylophora pistillata* raised from genetically identical genets [93]. Exposure to long-term pH-stress (pH 7.2) significantly increased mean methylation levels when compared to the control (pH 8.0). Methylation changes were observed in genes regulating cell cycle and body size. Enhanced DNA methylation at stressful pH was phenotypically accompanied by an increase in cell size and polyp size resulting in more porous skeletons. The paper demonstrates that environmental cues can concomitantly trigger changes of epigenetic marks on the DNA and phenotypic traits, suggesting a causal relationship between the two.

A good wild animal example of the exploitation of epigenetic mechanisms for environmental adaptation is the apomictic parthenogenetic New Zealand mud snail, *Potamopyrgus antipodarum*, in the western USA. All populations in this geographical area originated from a single clone that was introduced some 35 years ago, and, therefore, they are genetically largely identical [94]. They developed differences in shell shape between lakes and rivers, which were correlated with water current speed and associated with significant genome-wide DNA methylation differences [95]. Moreover, comparison of populations from a rural lake and two polluted urban lakes, which were characterized by high levels of phosphorous and faecal bacteria or high levels of heavy metals and organic xenobiotics, revealed differences in shell shape and allometric growth between lakes [96]. These differences were associated with numerous differentially methylated DNA regions (DMRs), suggesting adaptation to different environments and stressors by epigenetic mechanisms.

**Figure 2:**
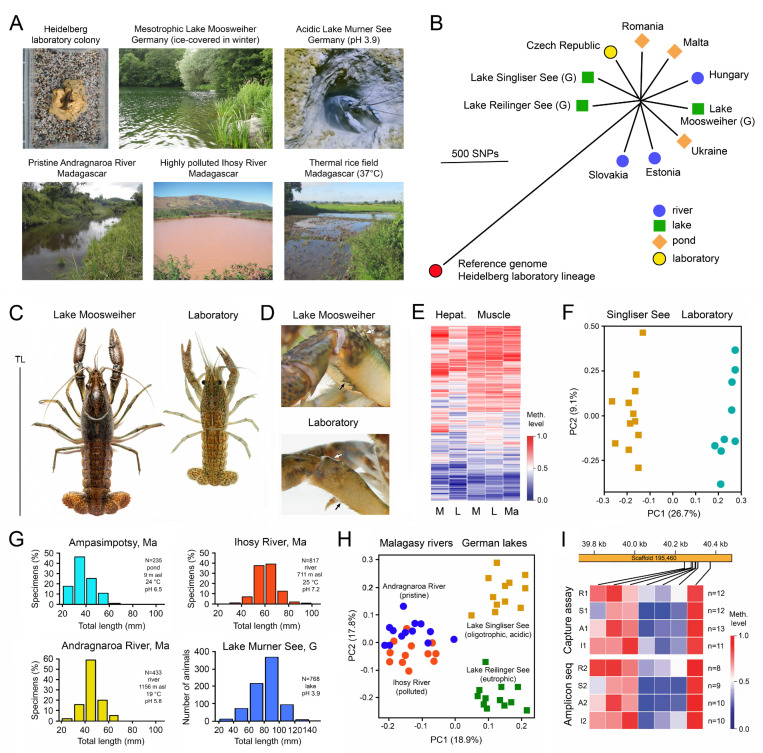
Phenotypic, genetic and epigenetic differences between differently adapted populations of marbled crayfish, *Procambarus virginalis*. (**A**) Examples of strikingly different marbled crayfish habitats. From Vogt et al. [104], Tönges et al. [105,108] and Andriantsoa et al. [107]. (**B**) Genetic differences between representatives from several European populations as determined by whole-genome sequencing. A descendant of the oldest known marbled crayfish aquarium lineage was used as a reference. G, Germany. Adapted from Maiakovska et al. [101]. (**C**) Maximum body size of laboratory raised and wild specimens from Lake Moosweiher (Germany), showing 30% bigger total length (TL) in the lake. From Vogt et al. [104]. (**D**) Chelipeds of specimens from the laboratory and Lake Moosweiher, showing bigger and sharper spines (arrows) in the wild specimen. From Vogt et al. [104]. (**E**) Comparative analysis of 697 variably methylated genes in the hepatopancreas and abdominal musculature of specimens from the laboratory (L), Lake Moosweiher (M) and a rice field in Moramanga, Madagascar (Ma). The heatmap shows differences in methylation patterns between individuals, particularly in the hepatopancreas. Adapted from Tönges et al. [105]. (**F**) Principal component analysis of samples from the laboratory and Lake Singliser See based on the average methylation of 361 variably methylated genes, showing clear separation of the populations. Adapted from Tönges et al. [105]. (**G**) Differences in population structure between pond, pristine mountain river and polluted lowland river in Madagascar and an acidic lake in Germany. Adapted from Andriantsoa et al. [107] and Tönges et al. [108]. (**H**) Principal component analysis of methylation of 122 genes separating four populations from rivers and lakes in Madagascar and Germany. Adapted from Tönges et al. [105]. (**I**) Persistent DNA methylation fingerprints of populations from Andragnaro River (A), Ihosy River (I), Lake Reilinger See (R) and Lake Singliser See (S) in consecutive years (1 and 2), exemplified for a small genic region of the hepatopancreatic DNA. The samples were collected at intervals of 12–21 months and analysed with two different methods. Adapted from Tönges et al. [105].

The apomictic parthenogenetic marbled crayfish, *Procambarus virginalis*, my favourite experimental animal, is another illustrative example of the adaptation of a genetically uniform species to different geographical regions and habitats. This all-female species evolved a few decades ago by autotriploidization from a single female of the Floridian slough crayfish, *Procambarus fallax* [97,98,99]. It appeared in the German aquarium trade in 1995 and was distributed from there across the world and frequently released, resulting in the establishment of wild populations in a broad spectrum of habitats in tropical to cold-temperate biomes in 22 European, African and Asian countries (Figure 2A). Coordinates and references are listed in [97,100,101].

High quality reference genomes of single individuals were assembled by Illumina and PacBio sequencing [102,103], and a genome-wide reference methylome was established by whole-genome bisulfite sequencing on an Illumina platform [47]. Comparison of whole genomes of 19 representatives from 15 wild populations in Europe and Madagascar with the reference genomes from laboratory specimens revealed very low genetic variation of a total of 16,564 single nucleotide polymorphisms (SNPs) in the 3.7 Gb genome (Figure 2B). About 74% of the SNPs were located in intergenic regions and only 4% in coding regions, among them very few non-synonymous variants that change amino acids in proteins [101,102].

Despite this genetic uniformity, wild marbled crayfish populations showed considerable phenotypic differences to laboratory-raised populations with respect to body size and coloration (Figure 2C), spination (Figure 2D) and body proportions [97,98,99,104]. These phenotypic differences were associated with DNA methylation differences in numerous genes (Figure 2E,F) [105], which were particularly prominent in the hepatopancreas, the main metabolic organ of crayfish [106]. Moreover, specimens reared in the laboratory for 6 months at either 10 °C or 20 °C exhibited significant differences in average methylation of 361 genes, providing experimental evidence for environmentally induced methylation changes within a single generation [105].

Significant differences were also found in size-frequency distribution between populations from different bio-climatic regions and habitats in Madagascar and Germany (Figure 2G) [107,108]. These water bodies include pristine and polluted rivers, oligotrophic and eutrophic lakes, ponds, rice fields, and cold, warm and acidic waters. Comparison of the methylation patterns of 122 selected, variably methylated genes in the hepatopancreas of specimens from pristine Andragnaro River and polluted Ihosy River in Madagascar and acidic, oligotrophic Lake Singliser See (no fishes present) and eutrophic Lake Reilinger See (many fishes present including species preying on crayfish) in Germany identified specific and highly localized DNA methylation signatures for each population (Figure 2H) [105]. These DNA methylation fingerprints remained stable over consecutive years (Figure 2I) [105].

Gene ontology analysis of the variably methylated genes revealed a significant enrichment of GTP-binding proteins, which transmit signals from the external world to the cells regulating various metabolic processes [105]. Enrichment was also recorded for proteins involved in regulation of transcription and translation, RNA metabolism, stress response and immune response. Since SNPs were absent from the differentially methylated genes investigated [105], the studies provide conclusive evidence for the independence of epigenetic variation from DNA sequence variation and the existence of epigenetic ecotypes.

Analyses of stable carbon ^13^C and nitrogen ^15^N isotopes of field samples from a Slovakian gravel pit, three German lakes, and the upper and lower stretches of a Hungarian stream revealed that marbled crayfish is also highly plastic with respect to food utilization, trophic position and niche width [109,110,111], despite genetic uniformity, but the epigenetic underpinning of these features has not yet been investigated.

Leung et al. studied the relative contributions of stochastic and environmentally induced epimutations to phenotypic variation in natural populations of the clonal, gynogenetic fish *Chrosomus eos-neogaeus* [80]. This all-female species occurs in North America in 14 clonal lineages originating from different hybridization events between the redbelly dace, *Chrosomus eos*, and the fine-scale dace, *Chrosomus neogaeus* [112]. Dating of hybridization events suggested an origin <50,000 years ago. Each hybrid lineage apparently originated from a single zygote and is genetically uniform with the exception of random mutations that accumulated over time.

The investigation of DNA methylation in *Chrosomus eos-neogaeus* lineages using methylation-sensitive amplification polymorphism (MSAP) revealed relative epigenetic similarity of individuals in a given lake but significant differences between lakes [21]. Analysis of DNA methylation in lineages from predictable and unpredictable environments (lakes versus headwater streams) in southern Quebec, Canada, identified the relative contributions of environmentally induced epigenetic variation (EEV) and stochastic epigenetic variation (SEV) to phenotypic variation [80]. Directional EEV was predominant in predictable environments, whereas risk-spreading SEV prevailed in unpredictable environments, suggesting that both strategies are differentially selected according to environmental uncertainty.

Differences in environmental effects on epigenetic variation between genetically diverse sympatric lineages showed that the epigenetic response is markedly influenced by the genotype [80]. Common garden experiments further revealed that the proportion of environmental effects can considerably change when clone members are transplanted to a new environment [80]. The example of *Chrosomus eos-neogaeus* demonstrates that EEV and SEV always occur together but have different weighting in different environments.

### 3.2. Case Studies with Plants

Plants are sessile and cannot evade unfavourable conditions by migration as most animals can do. Therefore, they may make particularly intense use of the possibility to rapidly produce phenotypic diversity by epigenetic mechanisms. The epigenetic mechanisms in plants are rather well investigated [50,113], but studies on epigenetic variation and associated phenotypic variation in natural populations are restricted to *Arabidopsis thaliana* and a few non-model species [114,115].

Shi et al. studied the dynamics of DNA methylation variation in Chinese populations of the clonal alligator weed, *Alternanthera philoxeroides*, by combining field monitoring with a multi-generation common garden approach [116]. The alligator weed is native to South America and has become invasive in many countries including China. The Chinese populations that cover a broad geographical range from tropical to temperate climates have a short history of less than 70 years and regenerate and spread entirely by asexual means. Using amplified fragment-length polymorphism (AFLP) and MSAP markers, the authors found very little variation in DNA sequence but substantial epigenetic population differences.

**Figure 3:**
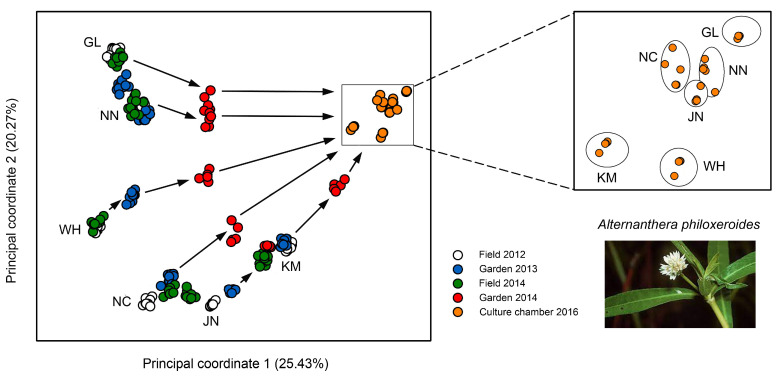
Variation of DNA methylation between and within differently adapted Chinese populations of clonal alligator weed, *Alternanthera philoxeroides*. Populations are indicated by two-letter code. The principal coordinate analysis shows samples from the field collected in subsequent years and the same samples after transfer to a common environment and then to a culture chamber. Zoom-in demonstrates that some of the DNA methylation differences between populations persisted for 10 asexual generations. Adapted from Shi et al. [116].

Epigenetic diversity was nearly 20-fold higher than genetic diversity and most likely resulted from a combination of environmentally induced and spontaneous epimutations [116]. In the field, these differences varied among locations and remained very stable across multiple years (Figure 3), pointing at the existence of epigenetic ecotypes. When transferred to a common environment, the DNA methylation patterns were maintained at first but then progressively eroded to a certain extent (Figure 3). However, after 10 asexual generations, there were still 38% of the original epiloci unchanged, indicating that a considerable proportion of the epimutations displayed at least medium-term stability.

Zhang et al. compared the magnitude of phenotypic variation and their heritability in *Arabidopsis thaliana* between genetic inbred lines (RILs), epigenetic recombinant inbred lines (epiRILs), and lines from broad-scale geographic collections of natural ecotypes in Eurasia and North America and a wild German population [117]. The epiRILs differed considerably in DNA methylation but only very little in DNA sequence, whilst the other lines differed in DNA methylation plus DNA sequence. The authors grew a total of 199 lines from genetic RILs, epiRILs and natural populations in a common environment and assessed their variation in growth, phenology and fitness.

Among-line phenotypic variation and heritability tended to be largest in natural ecotypes, but for some traits the variation in epiRILs was comparable to that in RILs and natural ecotypes [117]. Within-line phenotypic variation was generally similar in epiRILs, RILs and natural ecotypes. Under the assumption that the phenotypic variation in epiRILs is mainly caused by epigenetic differences and in RILs and natural ecotypes mainly by sequence variation, the results indicate that DNA methylation unrelated to genetic variation can create substantial heritable phenotypic variation.

Sammarco et al. assessed whether DNA methylation contributes to local adaptation and response to changed temperature in natural populations of asexually reproducing wild strawberry, *Fragaria vesca* [118]. Ramets were collected from Italy, Czechia and Norway, the southern, central and northern areas of the European range of the species. Within each country, three populations were collected along a gradient ranging from warmest to coldest mean annual temperatures. After clonal propagation and alteration of DNA methylation in half of the plants by the DNA methylation inhibitor 5-azacytidine, the clones were reciprocally transplanted to their home locality and the other two climatically distinct sites within the country of origin. At the end of the growing season, survival and aboveground biomass were recorded as fitness estimates.

The authors found evidence for local epigenetic adaptation in intermediate and cold populations in Italy and maladaptation of plants of the warmest populations in all countries [118]. Plants treated with 5-azacytidine showed either better or worse performance in their local conditions when compared to untreated plants. Application of 5-azacytidine also affected plant response to changed climatic conditions when transplanted to colder or warmer localities, and the response was country-specific. The authors concluded that DNA methylation can contribute to local adaptation in natural ecosystems, but its role may depend on the specific environmental conditions. They further argued that epigenetic adaptation might significantly help plants in coping with the ongoing climate change, because adaptation mediated by epigenetic variation occurs faster than by natural selection on genetic variants.

**Figure 4:**
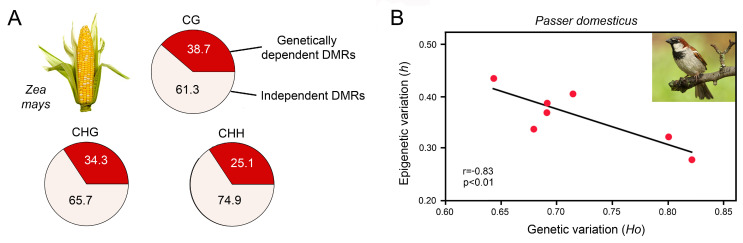
Genetic and epigenetic variation in genetically impoverished, sexually reproducing populations. (**A**) Dependence of DMRs on SNPs in CG, CHG and CHH contexts in 263 inbred genotypes of maize, *Zea mays*, showing that more than 60% of the epigenetic variation is uncoupled from genetic variation. Adapted from Xu et al. [119]. (**B**) Negative correlation of genetic and epigenetic variation in invasive populations of house sparrow, *Passer domesticus*, from seven Kenyan cities. Genetic variation was determined by microsatellite analysis and epigenetic diversity by MSAP. *h*, haplotype diversity; *Ho*, heterozygosity; p, probability value; r, Pearson correlation coefficient. Adapted from Vogt [33], compiled with data from Liebl et al. [120].

Xu et al. investigated DNA methylation at a population scale in 263 inbred genotypes of maize, *Zea mays* [119], which has a 17-fold larger genome when compared to *Arabidopsis thaliana*. The authors identified 8864, 9759 and 5075 DMRs for CG, CHG and CHH contexts, respectively, that were distributed across the 10 chromosomes. Genome-wide association analysis with high-density genetic markers revealed that over 60% of the DMRs were not tagged by SNPs (Figure 4A). Moreover, association analysis between DMRs and metabolic traits demonstrated that DNA methylation was associated with phenotypic variation in 156 traits. Many of these traits showed significant correlation with DMRs but not with SNPs, suggesting that epigenetic variation was not narrowly coupled with genetic variation.

### 3.3. Case Studies with Fungi, Protists and Bacteria

The production of epigenetic variation from the same genome is also documented for clonal fungal, protist and bacterial populations. A particularly well-known phenomenon is phenotypic switching, the rapid transition between two or more stable phenotypes [121]. This phenomenon is based on epigenetic mechanisms and of great importance in medicine, because it enables pathogens to enhance virulence and escape antibiotics treatment [6,121,122,123].

Kronholm et al. investigated phenotypic plasticity in filamentous fungus *Neurospora crassa* and found that this phenomenon is not governed by general plasticity genes [124]. It is, rather, context dependent and regulated by epigenetic mechanisms. For example, histone methylation at H3K36 contributed to the plastic response to high temperature and methylation at H3K4 to the response to pH alteration.

Khan et al. reviewed the effects of post-translational histone modifications on histone degradation in yeast *Saccharomyces cerevisiae* and *S. pombe*, which is an important regulatory mechanism of chromatin structure and gene expression change [125] contributing to phenotypic plasticity. They also addressed the role of histone modifications in controlling telomeric and centromeric silencing as examples of genetic loci that demonstrate epigenetic inheritance.

Protists are globally distributed in all ecosystems and play important roles in food webs and nutrient cycles. Mostly, they reproduce asexually, and, therefore, it remains enigmatic how the enormous diversity of protists was generated. Weiner and Katz argued that epigenetic processes such as chromatin modification and regulation by small non-coding RNAs that can rapidly modify genomes and gene expression states play important roles in driving phenotypic plasticity, differential adaptation and perhaps speciation of protists [126].

Huang et al. analysed global transcriptomic changes across 15 generations in the model diatom *Phaeodactylum tricornutum* exposed to elevated pCO_2_ by strand-specific RNA sequencing (ssRNA-seq) [127]. They found significant down-regulation of genes encoding histones and other proteins involved in chromatin structure. In contrast, the expression of transposable elements and genes encoding histone acetylation enzymes were significantly up-regulated. The authors also identified a series of long non-coding RNAs that specifically responded to elevated pCO_2_. They concluded that epigenetic mechanisms may play important roles in the acclimation of diatoms to environmental changes over short time scales and may thus contribute to long-term adaptation to ocean acidification.

Casadesús and Low reviewed the involvement of epigenetic mechanisms in the formation of distinct bacterial lineages, which is frequently observed during adaptation to harsh environments and the infection of animals and plants [128]. They came to the conclusion that the production of bacterial subpopulations is often controlled by epigenetic mechanisms that generate inheritable phenotypic diversity without altering the DNA sequence. The involved mechanisms range from relatively simple feedback loops to complex self-perpetuating DNA methylation patterns.

Pathogenic bacteria populations often contain a certain proportion of slowly growing or dormant cells that are genetically identical with the regularly growing cells. These dormant cells are highly tolerant to multiple antibiotics treatments. After treatment, when the regularly growing bacteria are killed, these so-called persisters start to multiply and establish a new population. There is conclusive evidence that epigenetic gene regulation and epigenetic long-term memory play a major role in persister formation [129].

Pathogenic microorganisms can develop sophisticated defence systems to survive in the host and become resistant to the host environment and to antibiotics treatment, promoting illness and mortality. Muhammad et al. reviewed the involvement of epigenetic changes in the development of antibiotics resistance in clinically relevant bacteria [130]. They reported on pronounced DNA methylation changes in the resistant bacteria, which sometimes led to the enhancement of their genetic mutation rates. Additionally, the bacteria induced histone modifications in the human host cells that promoted survival and long-term adaptation of the infectious bacterial populations.

Likewise, symbiotic microorganisms can not only change their own phenotypic traits in response to environmental cues by the use of epigenetic mechanisms but also influence gene expression in the host by changing its epigenome. An example is the microbiome in the human colon that can modify the epigenetic signatures of the neighbouring intestinal cells of the host and even the epigenome of the neurons in the hippocampus of the brain resulting in behavioural changes [131,132,133].

## 4. Environmental Adaptation of Genetically Impoverished Invaders with the Help of Epigenetic Mechanisms

Small invasive groups can conquer new environments never experienced before and proliferate despite paucity of genetic variation, which is known as the “genetic paradox of invasions” [22,23]. This section examines if epigenetic mechanisms can contribute to the adaptation of invasive populations to new environments and mechanistically explain the genetic paradox of invasions.

The studies described above with the obligatory parthenogenetic snail *Potamopyrgus antipodarum* and crayfish *Procambarus virginalis*, both of which have invaded many different habitats in the last decades, suggest that epigenetic mechanisms can be exploited for the invasion and adaptation of new environments. This also holds for small, sexually reproducing invasive groups with low genetic diversity.

An illustrative example is the house sparrow, *Passer domesticus*, that was introduced to Australia, Florida and Kenya ca. 160, 150 and 65 years ago, respectively. Since then, these populations have evolved significant phenotypic variation [120,134,135]. Analysis of microsatellite loci and MSAP of the Kenyan house sparrow populations revealed that they have low genetic diversity but high epigenetic diversity (Figure 4B) [120]. There was a significant negative correlation between genetic and epigenetic diversity and a positive correlation between epigenetic diversity and the inbreeding coefficient, suggesting that DNA methylation may help to overcome genetic barriers typically associated with invasions such as bottlenecks and inbreeding.

Schrey et al. compared the Kenyan and Floridian invasions genetically and epigenetically [134]. They found that the younger house sparrow populations in Kenya have less genetic diversity at multiple microsatellite loci but higher overall methylation when compared to their older Floridian relatives. However, some restriction fragments were more methylated in the Floridian population. The authors concluded that epigenetic variation may have compensated for low genetic variation during house sparrow invasions, facilitating colonization and establishment in new environments.

To investigate how fast epigenetic changes appear after invasion of a new environment, Hu et al. colonized eight small Caribbean islands of diverse habitat quality with brown anole lizard, *Anolis sagrei*, from a common source population [136]. Four days later they recaptured specimens from each island and determined genome-wide DNA methylation. They found that a significant proportion of the recorded epigenetic variation was explained by habitat quality. Cytosines were differently methylated in genes involved in signal transduction, circadian rhythm and immune response. The study demonstrates that epigenetic signatures can be changed already in the first days after colonisation and that these changes differ between habitats.

Mounger et al. examined the levels of genetic and epigenetic diversity in natural populations of *Rhizophora mangle* from the Gulf Coast of Florida by a reduced representation bisulfite sequencing approach [137]. *Rhizophora mangle* is a foundation species that occurs in coastal estuarine habitats throughout the neotropics. The authors found low genetic diversity but high epigenetic diversity in natural populations. In offspring grown in a common garden, about 75% of epigenetic differences was not explained by the maternal family, suggesting independence from genetic variation. Therefore, epigenetic variation could be an important source of phenotypic variation that helps to respond to challenging environments in genetically depauperate populations of this species. Further examples of the use of epigenetic variation for environmental adaptation in genetically depauperate invasive plants are found in recent reviews by Mounger et al. [138] and Rajpal et al. [139].

There are also emerging cases of the relationship between genetic and epigenetic diversity and the use of epigenetic mechanisms for environmental adaptation aside of clonal populations and genetically impoverished invasive populations. For example, using MSAP, Baldanzi et al. investigated genetic and DNA methylation variation among five differently adapted South African populations of the sandhopper *Talorchestia capensis* [140]. These populations showed high physiological differences in their response to environmental conditions. Population differentiation was higher for epigenetics than genetics with no clear geographical pattern. Four out of five populations showed significant negative correlations between epigenetic and genetic diversity. Likewise, members of the same population displayed greater variability in their epigenetic than their genetic profiles. These results show uncoupling between epigenetic and genetic variation and suggest that epigenetics is more responsive to local, site-specific environmental conditions than genetics and that individual differences in epigenetic profiles drive phenotypic variation. Within populations, epigenetics seems to offer a level of phenotypic flexibility beyond genetic constraint that allows rapid responses to unpredictable changes.

## 5. Ecological Implications of Epigenetic Diversity for Genetically Uniform Organisms and Possible Evolutionary Consequences

This review paper on the contribution of epigenetic variation to environmental adaptation focused on populations with near genetic identity with a bias to animals (Table 2). In these model systems, confounding effects of genetic variation are very small, and, therefore, the role of epigenetic mechanisms in producing phenotypic variation for ecological adaptation is much easier to recognize than in sexually reproducing populations. However, epigenetic mechanisms most likely also contribute to environmental adaptation in sexually reproducing organisms.

### 5.1. Capability of Epigenetic Mechanisms to Produce Phenotypic Variation for Environmental Adaptation

For a long time, it was taken for granted that the phenotypic variation required for environmental adaptation is exclusively based on genetic variation produced by mutation, recombination and drift [142,143,144]. In this concept, the environment was seen as the selector that decides which of the generated variants are best suitable for adaptation. The emergence of the phenotypic plasticity hypothesis [145,146] provided an alternative, suggesting that phenotypic variation can also be produced from the same genome in response to environmental signals without changing the DNA sequence. In this case, the environment is the inductor of phenotypic change. However, the molecular underpinning of phenotypic plasticity remained largely obscure. The detection of epigenetic mechanisms such as DNA methylation, histone modifications and non-coding RNAs [147] uncovered candidates that could mechanistically explain this phenomenon.

Laboratory and field studies, particularly with clonal populations, showed close associations between epigenetic variation and phenotypic variation, suggesting a functional relationship between the two [95,105,116,117]. However, studies that demonstrate cause–effect relationships between specific epigenetic signatures and phenotypic traits are still rare. A well-known animal example is the viable yellow A^vy^ mice (*Mus musculus*), in which a heritable yellow colour coat phenotype is caused by the activity and DNA methylation status of a retrotransposon driving abnormal expression of the *A^vy^* gene [148].

Another example is the influence of maternal care on the long-term stress response in the offspring of rat, *Rattus norvegicus* [149,150]. Differences in licking and grooming of pups by mothers over the first week after birth have pronounced effects on the stress response and social and reproductive behaviour of the filial generations. These behaviours cause epigenetic changes at the *glucocorticoid receptor* gene in the hippocampus of the brain, which encodes the receptor that binds glucocorticoid hormones to regulate the stress response. 

Epigenetic and phenotypic association studies and experiments, in which epigenetic marks are specifically changed, for example by CRISPR-Cas [151,152], could deepen our understanding of cause–effect relationships between epigenetic marks and phenotypic traits.

### 5.2. Habitat-Specific Epigenetic Signatures and the Existence of Epigenetic Ecotypes

Theoretically, the adaptation of clonal animals and plants to specific environments and the establishment of epigenetic ecotypes described above [105,116] could result from the repeated de novo production of similar, environmentally induced phenotypes in each generation or the transgenerational inheritance and selection of adaptive epigenotypes. The example of clonal alligator weed, *Alternanthera philoxeroides*, in which a certain proportion of the environment-specific epigenetic pattern was erased when transferred to a common environment and another proportion persisted for at least 10 generations [116], suggests that both processes may contribute to the establishment of epigenetic ecotypes. Recent papers and reviews on the inheritance of epigenotypes and related phenotypes indicate that the transgenerational inheritance of epigenetic signatures can play an important role in long-term environmental adaptation, indeed [153,154,155,156,157]. Performing cross-transplantation experiments and monitoring epigenetic fingerprints for dozens of generations could finally answer this question.

Epigenetic responses to environmental cues and subsequent phenotypic changes can affect many population members simultaneously in the first exposed generation, whereas beneficial genetic mutations and gene combinations first occur in single specimens and require many generations to be selected and become dominant in the population. Therefore, when combined with transgenerational epigenetic inheritance, the production of epigenetic phenotypic variation would be a perfect means to cope with transient environmental stressors and environmental changes [157]. If the triggering conditions should disappear in the lifetime of the exposed generation or the subsequent generation, the epigenetic marks and related phenotypes could be reverted to the old state, but when the eliciting conditions should become permanent, the epigenetic variants may persist and get selected, resulting in the establishment of epigenetic ecotypes.

It should be noted here that the rapid environmental adaptation of animals and plants can also be promoted by the microbiome via epigenetic mechanisms and fast accumulation of genetic mutations [158,159,160,161,162]. Animals and plants are metaorganisms that harbour numerous beneficial microorganisms on the body surface or in body cavities [158,161]. For example, a 70 kg reference man consists of about 3.0 × 10^13^ human cells and harbours 3.8 × 10^13^ bacteria [158,163]. Its gut microbiome consists of about 1000 bacterial species with 2000 genes per species, yielding an estimated 2 million genes. This number corresponds to 100 times the number of human genes, providing an enormous metabolic capacity [158]. Gene expression in these bacteria can change in response to different nutrients that enter the gut. So, if animals invade a new habitat with a different food spectrum, expand their range or change their feeding behaviour, the microbiota can rapidly adjust to this situation and help the host to rapidly adapt to the new conditions.

### 5.3. Epigenetic Variation as an Explanation of the General-Purpose Genotype and Invasion Paradox

The well documented adaptation of clonal animals and plants to a wide range of geographical regions and habitats was often explained with the general-purpose genotype hypothesis [19,20,21,164,165]. General-purpose genotypes are thought to be able to produce different phenotypes across a wide range of environmental conditions that maintain high fitness regardless of habitat [19]. But how can they do this? Massicotte and Angers measured different DNA methylation patterns in differently adapted populations of the clonal gynogenetic fish *Chrosomus eos-neogaeus* and concluded that DNA methylation may be among the molecular mechanisms underpinning the general-purpose genotype [21]. The case study with the highly invasive marbled crayfish [105] supports this view.

The ability of general-purpose genotypes to produce broad ranges of phenotypes in different environments must anyhow be encoded in the genome. Apparently, such genotypes do not possess enigmatic “general plasticity genes” [124]. Rather, they seem to own well developed machineries that can change the epigenetic code on the DNA and histones in response to a broad spectrum of environments. Additionally, they may possess a particularly large set of environment-responsive, gene regulatory non-coding RNAs.

Invasive species are regarded as a major factor for the loss of biodiversity and modern environmental damages, causing costs of several hundred billion USD per year [166]. Therefore, it is of prime importance to understand how invasive species generate enough phenotypic variation to survive in new environments and extend ranges [167]. Invasive groups often consist of a few specimens only, and, therefore, genetic diversity is low in the early period of invasions, even in sexually reproducing invaders. It is probably not sufficient to generate the phenotypic diversity required to cope with the challenges of the invaded environment. Nevertheless, small invasive groups can conquer environments never experienced before, occupy different niches and establish vivid populations in relatively short time scales of some years or decades, which seems paradoxical [22,23].

Environmental adaptation by epigenetic mechanisms may provide an explanation for this invasion paradox [33,138,168,169]. Particularly the first steps of invasion, the survival of the invading specimens and the establishment of a founder population, seem to depend much on epigenetic mechanisms. Bed-hedging stochastic epimutations may broaden the phenotypic spectrum of successful invaders already before the invasion begins, serving as a kind of predisposition. Short- and medium-term adaptation to the newly invaded habitat is thought to be fostered mainly by directional epimutations triggered by the new environmental cues. The generation of phenotypic diversity by genetic variation may become relevant only in the long term, when enough genetic mutations are available.

### 5.4. Relevance of the Production of Epigenetic Variation for the Ecology of Asexually Reproducing Organisms

Asexually reproducing organisms generate much less genetic variation than sexual reproducers due to the absence of meiotic recombination. Therefore, they likely depend more on the generation of phenotypic variation by epigenetic mechanisms. The experiments and field work with clonal organisms and sexually reproducing populations with low genetic diversity described above demonstrate that epigenetic variation can, to some extent, compensate for the lack of genetic variation when adapting to different environments.

In animals, sexual reproduction is predominant but asexual reproduction is widespread in several sessile taxa including sponges, cnidarians, bryozoans and ascidians, and some parasitic groups such as trematodes and cestodes. Moreover, common agricultural pests such as aphids and key species of aquatic food chains such as water fleas often reproduce by facultative parthenogenesis [20,170,171,172,173]. In these animals, epigenetic variation apparently underpins the establishment of epigenetic ecotypes, fosters invasiveness and helps parasites to adapt to their hosts.

Plants and multicellular fungi mostly combine sexual and asexual reproduction, and the balance between the two varies widely between and within species [174,175]. For example, clonal or vegetative reproduction was found in 66.5% of the central European vascular plants, and in 112 vegetation types investigated, the frequency of clonal plants exceeded that of nonclonals [176]. Like in animals, epigenetic variation is supposedly the basis of the establishment of epigenetic ecotypes, enhances the probability of successful invasions and helps parasites and pathogens to adapt to their hosts [116,117,138,177,178].

In bacteria, clonal reproduction is the rule, and in protists and unicellular fungi, it is predominant. In these microorganisms, epigenetic variation is involved in adaptation to natural environments [128] and possibly in generation of species diversity [126], as will be explained in more detail in the following section. In pathogenic microorganisms, epigenetic variation plays an important role in the enhancement of virulence and resistance against antibiotics [122,129].

### 5.5. Evolutionary Potential of Epigenetically-Based Phenotypes and Epigenetic Ecotypes in Clonal Organisms

In this section, I will discuss whether the generation of phenotypes and ecotypes by epigenetic mechanisms can influence the evolution and diversification of asexually reproducing or otherwise clonal organisms. The evolutionary potential of epigenetic signatures and related phenotypic traits is highly dependent on whether they are inherited across generations and genetically integrated in the long-term. Empirical research revealed that epigenetic marks and associated phenotypes can be transgenerationally inherited, indeed [153,154,155,156,157]. However, most studies in animals and plants were run for 1–3 generations only [179]. Exceptions include some studies with water flea *Daphnia pulex*, nematode *Caenorhabditis elegans*, tale cress *Arabidopsis thaliana* and the unicellular green alga *Chlamydomonas reinhardtii*, which were run for 15, >20, 30 and ~200 generations, respectively [180,181,182,183]. New studies over many more generations are necessary to obtain a better picture.

Experiments have also shown that the extent of epigenetic inheritance depends very much on trait and condition. Modelling revealed that long-term effects of epimutations are strongly determined by their stability and fitness effects relative to genetic mutations [184]. Sperm seems to be particularly effective in transmitting epigenetic information to the next generation via conservation of DNA methylation, histone modifications and non-coding RNAs [185,186].

In a well-adapted population living in a constant environment it makes little sense to inherit epigenetic variants over many generations because it may incur costs but provide no advantage. However, if the environment changes from one stable condition to another stable condition, then new and better suited epigenetic variants may be selected and stably inherited. Interestingly, *Caenorhabditis elegans* was shown to possess a timing mechanism based on non-coding RNAs that controls the duration of transgenerational epigenetic inheritance [187], which may help to maintain or reset an epigenetic phenotype.

In sexually reproducing species, beneficial epigenetic phenotypes can be fixed on the long term by genetic assimilation, a process by which a phenotype originally produced in response to environmental signals is later taken over by the genotype via selection on random genetic mutations with similar phenotypic effects [188,189]. An alternative, more directional mechanism, which would also be applicable to asexually reproducing species, is the transformation of epimutations with phenotypic effects to corresponding genetic mutations [190]. Methylated CpGs mutate into TpGs in a 10–50-fold higher probability than unmethylated CpGs [191]. The biological significance of such facilitated mutations is well documented for bacteria. Methylated cytosines were identified as hot spots for cytosine-to-thymine mutations that changed crucial fitness traits such as antibiotics susceptibility [122]. In animals, phenotypically relevant mCpG-to-TpG transitions can occur in relatively short periods of time, as demonstrated for domesticated European seabass, *Dicentrarchus labrax*. Several methylated CpGs that were established from unmethylated CpGs in the second generation of domestication in response to the new culture conditions appeared as TpGs after 25 generations [192]. This way, a principally reversible, epigenetically determined phenotype could become a permanent, genetically encoded phenotype. The long-term genetic integration of epigenetic phenotypes may lead to the transformation of epigenetic ecotypes into classical, genetically determined ecotypes.

According to traditional belief, clonal lineages are dead ends of evolution because of the absence of genetic recombination, the most effective mechanism to create new phenotypes. However, the bdelloid rotifers that lived without sex for 40 million years yielded four families, 18 genera and 360 species [193]. A similar diversity evolved in some obligatory parthenogenetic freshwater ostracod groups, which exist for more than 100 million years without sex [173]. The diversity of such evolutionarily successful asexuals is usually explained with the temporary appearance of sexually reproducing individuals, the separate origin of clones from different sexual ancestors, and hybridization between asexual females and males from related species, but there is little proof for any of these hypotheses.

**Figure 5:**
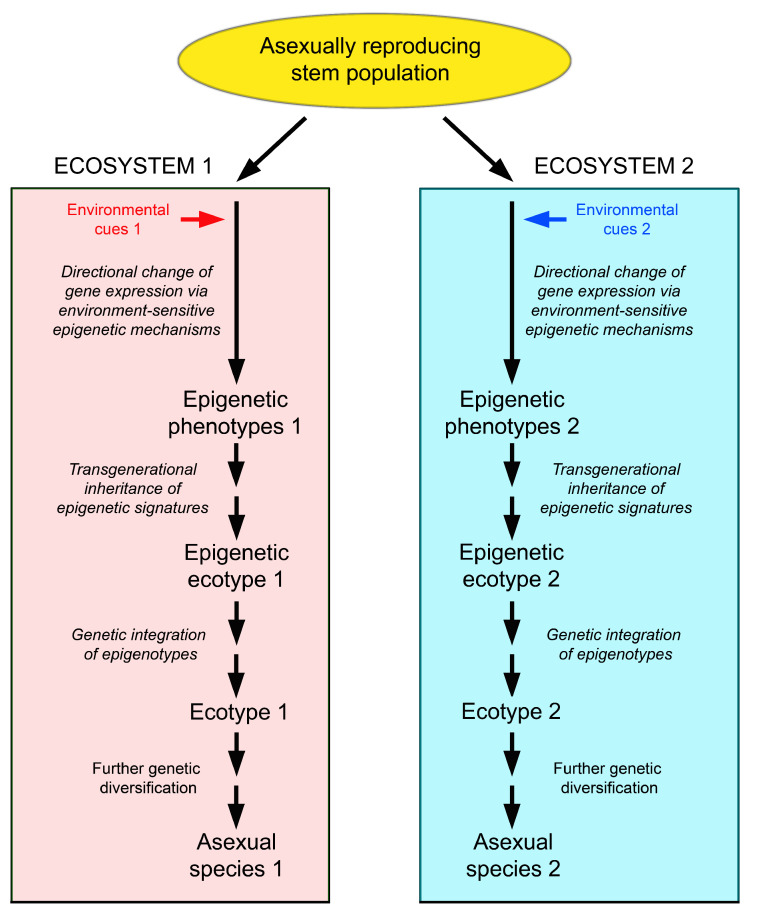
Scenario of speciation in asexually reproducing organisms via epigenetic phenotypes and epigenetic ecotypes. Different epigenetic ecotypes arise from a genetically uniform source population by invasion of different ecosystems, the generation of habitat-specific phenotypes by environmentally induced epigenetic changes, and the transgenerational inheritance and selection of these phenotypes. Under favourable conditions, the epigenotypes may be genetically integrated, and the epigenetic ecotypes may thus transform into classical, genetically diverse ecotypes, which can finally evolve to different species.

An alternative explanation might be speciation via the following sequence: generation of different phenotypes in different environments by epigenetic mechanisms → establishment of epigenetic ecotypes in the short and medium term → conversion of epigenetic ecotypes into classical, genetically determined ecotypes in the long term by conversion of epimutations into genetic mutations → evolution of these ecotypes into different species by classical evolutionary mechanisms [194] (Figure 5).

The transition of short-term and reversible acclimation by epigenetic mechanisms to long-term and permanent evolutionary adaptation by genetic mechanisms may be illustrated by the adaptation to high altitude. Altitude acclimation in humans is characterized by complex physiological responses, which include the cardiovascular, haemopoietic, respiratory and metabolic systems. Childebayeva et al. collected DNA samples from participants trekking to Everest Base Camp to identify DNA methylation changes associated with incremental altitude ascent [195]. They compared two altitudes by determining genome-wide DNA methylation levels: baseline 1400 m at day 0 and elevation 4240 m at day 7. The authors revealed 2873 differentially methylated positions (DMPs) and 361 differentially methylated regions (DMRs), including significant sites in the hypoxia inducible factor (HIF) and the renin–angiotensin system (RAS) pathways. Pathway enrichment analysis identified 95 affected pathways including regulation of glycolysis, haematopoietic stem cell differentiation and angiogenesis.

The Tibetans have populated the highlands of the Himalaya about 7000 years ago corresponding to ~280 generations [196]. They are now genetically adapted to this special environment [196,197,198], a process that probably started with epigenetically-based acclimation and included the conversion of epimutations into functionally corresponding genetic mutations. Many of the genes associated with high altitude adaptation are members of the HIF pathway. Products of this pathway include vascular endothelial growth factors, erythropoietin and glycolytic enzymes that respond to lack of oxygen by increasing oxygen delivery via numerous cellular and systemic changes or switching to anoxic metabolic pathways.

Another example of the transition of high-altitude acclimation to adaptation is the Andean house wren, *Troglodytes aedon* that inhabits a range of altitudes from sea level to above 4500 m. This bird exhibits changes in the oxygen affinity of haemoglobin with increasing altitude, which is attributable to an ancestral CpG site that mutated to CpA resulting in a non-synonymous amino acid substitution. The frequency of the mutated allele linearly increases with altitude, such that populations at high altitudes have fixed the mutated CpA, whereas low-altitude populations have retained to CpG [198]. Likely, environmentally induced methylation of CpG and their facilitated mutation to CpA has played a role in this process.

## 6. Short Digression into the Implications of Epigenetic Variation for Environmental Adaptation in Genetically Diverse Animals, Plants and Microorganisms

The relative contribution of genetic and epigenetic variation to the production of phenotypic diversity for environmental adaptation seems to depend on several factors, including mode of reproduction, lifestyle and life history parameters. Since these factors vary significantly between and within animals, plants and microorganisms, different higher taxa and species may profit differently from genetic and epigenetic mechanisms. The first parameter to be discussed is mobile versus sessile lifestyle. Mobile organisms can evade alterations of the environmental conditions by migration, but sessile organisms cannot. Animals are mostly vagile and plants are sessile. Therefore, epigenetic mechanisms may be particularly intensely exploited for environmental adaptation by plants and sedentary animals such as the Porifera, Cnidaria, Bryozoa, Bivalvia, Cirripedia, Tunicata and Pelmatozoa. However, because high values of DNA methylation were also found in numerous vagile animals from different groups [37], all animals may to some degree use epigenetic variation for environmental adaptation including range expansion and climate change [199].

In asexually reproducing species, phenotypic variation is almost exclusively based on epigenetic variation [9,32,99], whereas in sexual reproducers, it relies on both genetic and epigenetic sources. In the latter, genetic variation may often be predominant, but studies on the relative contribution of genetic and epigenetic mechanisms to phenotypic variation are scarce.

The degree of genetic diversity that can be generated in a population or species is not only dependent on the mutation rate and mode of reproduction but also on population size and generation time. Species with low individual numbers and long generation times produce much less genetic variation when compared to species with high population numbers and short generation times. Bacteria usually have extremely high population densities and very short generation times, and, therefore, they can rapidly produce high degrees of genetic variability by genetic mutation. For example, *Escherichia coli* can reach population densities of >10^9^ cells per mL culture medium, and the total number of symbiotic bacteria in the human colon was estimated to 3.8 × 10^13^ [163]. Under optimal conditions, *E. coli* can divide every 20 min in the laboratory but *Syntrophobacter fumaroxidans* doubles only every 140 h. In their host, human pathogenic bacteria vary in generation time from 1.1 h in *Vibrio cholerae* to 25 h in *Salmonella enterica* [200]. Plants and animals have much longer generation times. In plants they vary between 1 and 40 years [201] and in animals between a few days in water fleas to about 15 years in killer whale *Orca orca* [202,203].

The occurrence of striking differences in generation time and population size between members of the same animal group is illustrated for mammals with the examples of elephant and rat. The African elephant *Loxodonta africana* reproduces for the first time at a mean age at 13.8 years [204] and has a world-wide population of less than 500,000 individuals, suggesting that their potential to generate phenotypic variation by random mutation, recombination and drift is rather limited. In contrast, the world population of rat, *Rattus norvegicus*, is estimated to 7 billion individuals. Their generation time is only 3–4 months [205], enabling production of genetic diversity in a much higher rate.

The degree of epigenetic diversity that an organism can produce is dependent on its epigenetic machinery, the ratio of spontaneous forward and backward epimutations and the induction and erasure of environmentally induced epimutations. In populations, new environmentally induced and epigenetically mediated phenotypes can spread rapidly, because many population members can be affected synchronously in the first exposed generation. This is in contrast to new phenotypes arising from random genetic mutations that start with single individuals and require multiplications through numerous generations to get established.

The considerations presented in this section suggest that asexually reproducing, sessile and long-lived organisms with long generation times are the main benefiters of environmental adaptation by epigenetic mechanisms. Therefore, the relevance of epigenetics for adaptation should, in general, be highest for plants, intermediate for animals and lowest for microorganisms.

## 7. Conclusions

The adaptation of genetically uniform organisms to different environments is a special but particularly insightful perspective on ecoepigenetics (Box 1). It demonstrates that epigenetic mechanisms can produce many different phenotypes from the same genotype, that epigenetic ecotypes can be formed in short periods of time, and that epigenetic mechanisms are among the molecular players underpinning the general-purpose genotype and the invasion paradox. The presented examples of asexually reproducing animals, plants and microorganisms indicate that the production of epigenetically mediated phenotypic variation for environmental adaptation is a universal biological principle.

Box 1 **Ecoepigenetics knowledge obtained with clonal organisms and main open questions.**

- Epigenetic mechanisms can produce phenotypic variation from the same DNA sequence.

- Epigenetic variation helps to cope with short- to medium-term environmental challenges.

- Epigenetic variation is used to produce different epigenetic ecotypes in genetically uniform organisms.

- Epigenetic variation likely underpins the general-purpose genotype.

- Epigenetic variation is suitable to explain the invasion paradox.

- Epigenetic variation may be the starting point of the evolution of species diversity in asexuals.

- Is transgenerational epigenetic inheritance involved in the production of epigenetic ecotypes?

- Can epigenetic ecotypes evolve into classical genetically based ecotypes and, finally, into different species?

The generation of epigenetically mediated phenotypic variation seems to be of different importance in different biological contexts. In asexually reproducing species and lineages, it is probably indispensable for adaptation to different environments. In genetically impoverished groups of sexually reproducing invaders, the generation of epigenetic variation seems to be particularly important for the survival and establishment of the first generations in the new environment. In genetically more diverse sexually reproducing populations, epigenetic variation supposedly supplements genetic variation and is particularly helpful in coping with environmental stress, adaptation to rapid environmental changes, and range expansion. Asexual reproducers, sessile taxa and long-lived species seem to particularly profit from this mode of generation of phenotypic diversity.

The epigenetically mediated generation of reversible phenotypic variation may ultimately end up in genetically fixed and irreversible phenotypic variation. This may be achieved via genetic assimilation and/or the conversion of epimutations to genetic mutations with similar phenotypic effects. This way, epigenetics-based phenotypic plasticity and acclimation may be the first step towards genetics-based evolutionary adaptation and speciation, explaining the unexpectedly high diversity in some asexually reproducing higher taxa. However, much work remains to be performed to test this hypothesis experimentally.

The consideration of epigenetic mechanisms for the generation of phenotypic plasticity may significantly broaden our understanding of environmental adaptation, the response of organisms to environmental challenges, biological invasions, range expansion, and the evolution of asexual organisms.

## Figures and Tables

**Table 1:** Mechanisms for generating phenotypic diversity in populations.

Genetic Mechanisms (Act by DNA Sequence or Frequency Change)	Epigenetic Mechanisms (Act without DNA Sequence Change)
Mutation	DNA methylation
Recombination	Histone modifications
Genetic drift	Non-coding RNAs
Gene flow	Polycomb/Trithorax system
Alternative splicing ^1^	mRNA editing
	mRNA modifications

^1^ produces alternative mRNAs from the same DNA without changing its sequence.

**Table 2:** Selected case studies and review articles on the association of epigenetic variation and environmental adaptation in genetically uniform populations.

Species	Epigenetic Mechanism	Reference
Animals		
*Stylophora pistillata* ^a^	DNA methylation	Liew et al. [93]
*Procambarus virginalis* ^a^	DNA methylation	Tönges et al. [105]
*Potamopyrgus antipodarum* ^a^	DNA methylation	Thorson et al. [95]
*Potamopyrgus antipodarum* ^a^	DNA methylation	Thorson et al. [96]
*Chrosomus eos-neogaeus* ^a^	DNA methylation	Massicotte and Angers [21]
*Chrosomus eos-neogaeus* ^a^	DNA methylation	Leung et al. [80]
*Anolis sagrei* ^b^	DNA methylation	Hu et al. [136]
*Passer domesticus* ^b^	DNA methylation	Schrey et al. [134]
*Passer domesticus* ^b^	DNA methylation	Liebl et al. [120]
Plants		
*Alternanthera philoxeroides* ^a^	DNA methylation	Shi et al. [116]
*Fragaria vesca* ^a^	DNA methylation	Sammarco et al. [118]
*Taraxacum officinale* ^a^	DNA methylation	Wilschut et al. [141]
*Arabidopsis thaliana* ^c^	DNA methylation	Zhang et al. [117]
*Zea mays* ^c^	DNA methylation	Xu et al. [119]
*Rhizophora mangle* ^b^	DNA methylation	Mounger et al. [137]
*Various species* ^b^	Various mechanisms	Mounger et al. [138]
*Various species* ^b^	Various mechanisms	Rajpal et al. [139]
Fungi		
*Neurospora crassa* ^a^	Histone modifications	Kronholm et al. [124]
*Candida albicans* ^a^	Histone modifications	Rai et al. [6]
*Saccharomyces spec.* ^a^	Histone modifications	Khan et al. [125]
Protists		
*Various species* ^a^	Various mechanisms	Weiner and Katz [126]
*Phaeodactylum tricornutum* ^a^	Histone modif., ncRNAs	Huang et al. [127]
Bacteria		
*Various species* ^a^	DNA methylation	Casadesús and Low [128]
*Escherichia coli* ^a^	DNA methylation	Ghosh et al. [122]
*Escherichia coli* ^a^	DNA methylation	Riber and Hansen [129]
*Various species* ^a^	DNA methylation	Muhammad et al. [130]

^a^ Asexually reproducing; ^b^ sexually reproducing, genetically depauperate invader; ^c^ sexually reproducing, inbred.

## Data Availability

Not applicable.
